# Core Epithelial‐to‐Mesenchymal Transition Gene Signature Predicts Metastasis and Poor Survival in Synovial Sarcoma

**DOI:** 10.1002/cam4.71643

**Published:** 2026-02-25

**Authors:** Megan Jagosky, Colin Anderson, Nury Steuerwald, Jenny Chen, Anderson O' Brien Cox, HsihTe Yang, Guangxu Jin, Alicia Hamilton, Mathew Smith, Sharvil Desai, Johann Hsu, Wei Zhang

**Affiliations:** ^1^ Department of Solid Tumor Oncology Atrium Health Levine Cancer Institute Charlotte NC USA; ^2^ Allina Health Orthopedics and Allina Health Cancer Institute, Allina Health Minneapolis MN USA; ^3^ The Molecular Biology and Genomics Laboratory Levine Cancer Institute, Atrium Health Charlotte NC USA; ^4^ Department of Biostatistics and Data Sciences Levine Cancer Institute, Atrium Health Charlotte NC USA; ^5^ Bioinformatics Shared Resource, Comprehensive Cancer Center, Wake Forest University School of Medicine Winston‐Salem NC USA; ^6^ Department of Cancer Biology Wake Forest University Winston Salem NC USA

**Keywords:** EMT, gene expression, mesenchymal, metastasis, prognostic biomarkers, synovial sarcoma

## Abstract

**Background:**

Synovial sarcoma (SS) is a rare soft tissue malignancy comprising 5%–10% of all sarcomas, typically affecting young adults. It is characterized by a pathognomonic SS18–SSX gene fusion, with variable histologic subtypes demonstrating epithelial and mesenchymal features. This study investigates the association between epithelial‐to‐mesenchymal transition (EMT) gene expression and metastatic potential in SS.

**Methods:**

A retrospective, single‐institution analysis was conducted using primary tumor specimens from 21 treatment‐naïve SS patients. RNA sequencing was performed on formalin‐fixed paraffin‐embedded (FFPE) tissue to assess gene expression profiles. EMT scores were calculated using three independent methods: Hallmark EMT gene set from GSEA, the 76‐gene EMT signature (76GS), and Kolmogorov–Smirnov (KS) testing. Patients were categorized into metastatic and nonmetastatic cohorts, and phenotype‐specific survival analyses were conducted.

**Results:**

Tumors from patients who developed metastases showed significant enrichment of EMT‐related genes (GSEA NES 1.71, *P* = 0.025), oxidative phosphorylation, and immune‐related pathways. EMT scores were consistently higher (more mesenchymal) in metastatic tumors across all scoring methods. Mesenchymal phenotype was associated with significantly worse overall survival (GSEA log‐rank *P* = 0.0009).

**Conclusions:**

This study demonstrates a correlation between mesenchymal gene expression signatures and increased metastatic risk in SS. EMT status, derived from primary tumor profiling at diagnosis, may serve as a potential prognostic biomarker. These findings support further investigation into EMT as a stratification tool for tailoring treatment intensity and surveillance strategies in SS.

## 1 Background

Synovial sarcoma (SS) accounts for approximately 5%–10% of all soft tissue sarcomas and affects a predominantly younger population with median age of onset of 39 [1]. It is thought to originate from primitive, multipotent mesenchymal cells [2]. Oncogenesis is related to the pathognomic gene fusion stemming from translocation *t*(X;18)(p11;q11) that 95% of synovial sarcoma cases harbor [3]. This translocation involves the gene SS18 on chromosome 18 and an SSX gene family member on chromosome X, the most common being SSX1, followed by SSX2 and rarely SSX4. This translocation forms the SS18–SSX protein that serves as a transcription cofactor shown to interfere with the SWI/SNF chromatin remodeling complex, polycomb repressor complex, and canonical Wnt pathway [4]. The downstream effects have not been fully expounded.

SS cells harbor phenotypic plasticity, demonstrating three morphologic variants: monophasic (homogenous spindle morphology), biphasic (spindle and epithelial morphology), or poorly differentiated [5]. The fusion partner is related to the histologic subtype with the biphasic morphology more often harboring the SS18–SSX1 fusion transcript, whereas monophasic morphology more often has the SS18–SSX2 fusion transcript [6].

Most patients with synovial sarcoma present with early‐stage disease with only 6%–18% having de novo metastasis. However, a significant 50% of patients are estimated to develop metastasis at some point in their disease course. Patients presenting with metastatic disease have a 5 year overall survival of 10% compared to 76% for those who have localized disease at time of diagnosis [7]. Predicting which patient will develop metastatic disease is challenging. Given synovial sarcoma likely originates from multipotent mesenchymal cells with biphenotypic morphology seen, it is of interest to determine if epithelial‐to‐mesenchymal transition impacts risk of metastasis.

Epithelial to mesenchymal transition (EMT) and its reverse, mesenchymal to epithelial transition (MET) occur naturally as a part of normal tissue development [8]. Cells can reorganize their cytoskeleton and reprogram their behavior to demonstrate features of epithelial or mesenchymal phenotypes. The epithelial phenotype is marked by cohesive cell‐to‐cell interactions that can proliferate readily. The mesenchymal phenotype is marked by flexibility, changing to a spindle shape, breaking cell‐to‐cell interactions, and dynamic motility [9]. EMT/MET has been widely studied in various cancer types, with a handful of studies demonstrating this process in sarcoma [10]. While sarcoma is predominantly a mesenchymal malignancy, certain subtypes may harness the MET pathway, developing intratumor heterogeneity and being more apt to transition to the epithelial phenotype and back. This would allow greater flexibility for metastasis and re‐establishment of a tumor in a metastatic deposit that would need to adapt to its microenvironment.

A large, single‐institution, retrospective analysis was conducted on banked primary synovial sarcoma specimens at our institution to further elucidate differences in gene expression pathways between patients who developed metastasis and those who did not. Additionally, the study sought to evaluate the real‐world impact of EMT/MET and phenotype heterogeneity in SS behavior.

## 2 Methods

This retrospective, single‐institution study included tissue specimens acquired from patients during their routine standard of care work‐up and treatment at Levine Cancer Institute, Charlotte, North Carolina. The tissue specimens were acquired from the patient's primary site of the disease. Specimens from patients that had been exposed to prior chemotherapy or radiation were excluded. Histologically confirmed synovial sarcoma cases were included from patients aged 16 and older. Patient demographics and clinical characteristics were acquired from patients' electronic medical records. This study was approved by the Institutional Review Board at Atrium Health (LCI‐SAR‐SYN‐001R). Due to the retrospective nature of this study and minimal risk to patients, informed consent was determined to be unnecessary.

The tissue specimens were stored in formalin‐fixed paraffin‐embedded (FFPE) blocks prior to the study. These blocks were sectioned and hematoxylin and eosin (H&E) stained to allow assessment by the pathologist for tumor cellularity. Ten‐micron sections were cut from those specimens deemed adequate for analysis to be used in genomic testing. Genomic deoxyribonucleic acid (DNA) and total ribonucleic acid (RNA) were simultaneously purified from FFPE tissue sections using the AllPrep DNA/RNA FFPE kit (Qiagen) as directed by the manufacturer. Briefly, paraffin is removed from the specimen prior to lysis with proteinase K. The sample is centrifuged to obtain an RNA‐containing supernatant and DNA‐containing pellet. RNA or DNA is isolated by spin column centrifugation prior to elution.

Transcriptome profiling was performed by RNA sequencing. RNA integrity was evaluated by an Agilent 2100 Bioanalyzer profile (Agilent, Santa Clara, CA, USA). The percentage of RNA fragments greater than 200 nucleotide (nt) fragment distribution value (DV200) was used to assess FFPE RNA quality. Next generation sequencing libraries were prepared using an Illumina RNA Exome kit (Illumina) as directed. Briefly, the RNA was fragmented using divalent cations under elevated temperature following purification. Reverse transcription of the cleaved RNA fragments was primed with random hexamers during first‐ and second‐strand cDNA synthesis. The 3′ ends were adenylated prior to ligation of the sequencing adapters. The adapter‐ligated fragments were enriched by polymerase chain reaction (PCR) amplification and quantified. Probes specific to the coding regions of the transcriptome were used to bind targeted regions of DNA. Streptavidin magnetic beads were used to capture hybridized probes to create a sequencing library. Hybridization and capture were repeated a second time to ensure high specificity of the captured regions. The libraries were sequenced on an Illumina NextSeq 2000 using a NextSeq 2000 200 cycle flowcell.

Two cohorts of patients were established including those that had metastasis develop at some point before diagnosis or after diagnosis and those that did not. The primary objectives were whether expression of EMT genes contributed to risk of metastasis and whether there was a difference in phenotype between the cohorts. Three methods were used to determine an EMT score for each cohort using (1) core enrichment genes from the gene set enrichment analysis (GSEA) Hallmark EMT gene set by additionally including CDH1 gene as the reference, (2) a previously published default 76‐gene signature (76GS) for EMT, and (3) a two‐sample Kolmogorov–Smirnov (KS) test [11, 12, 13].

The EMT scores for methods 1 and 2 were calculated based on the weighted sum of expression levels, with each gene's weight being the correlation coefficient between its expression level and that of epithelial marker E‐cadherin (CDH‐1) in the dataset. Negative scores were interpreted as mesenchymal (M) phenotype, whereas the positive scores were epithelial (E) phenotype.

The EMT score for method three used a two‐sample KS test which compared the cumulative distribution functions (CDFs) of E and M signatures. The score was determined through hypothesis testing with a null hypothesis as no difference in CDFs of M and E signatures. The alternative hypotheses were: (1) the CDF of M signature is greater than that of E signature and (2) the CDF of E signature is greater than that of M signature. The sample with a positive EMT score is classified as M while a negative EMT identifies an E phenotype.

Lastly, differences in survival between E and M phenotypes were determined by dividing samples binomially based on their EMT scores with log‐rank testing and cox proportional hazards model testing performed. Given the exploratory nature of this survival analysis and the rarity of synovial sarcoma, the statistical significance was evaluated using an alpha level of 0.10. Because the study is retrospective and exploratory, a formal power analysis was not performed.

To explore whether EMT phenotype was independently associated with overall survival, we performed Cox proportional hazards regression. Candidate covariates were selected a priori based on clinical relevance and included sex, race, ethnicity, age at diagnosis, smoking status, tumor size, stage at diagnosis, mitotic count, tumor necrosis, and receipt of perioperative chemotherapy and radiation. Because the cohort was small and the number of deaths was limited (3 events among 21 patients), we first fit univariable Cox models. Variables with *P* < 0.10 (EMT phenotype, age at diagnosis, and tumor size) were then entered into a multivariable Cox model. To address complete or quasi‐complete separation (e.g., no deaths among patients with an epithelial EMT phenotype or in certain clinical subgroups) and reduce small‐sample bias, we used Firth's penalized likelihood approach and restricted the analysis to complete cases (*n* = 15).

## 3 Results

**FIGURE 1:**
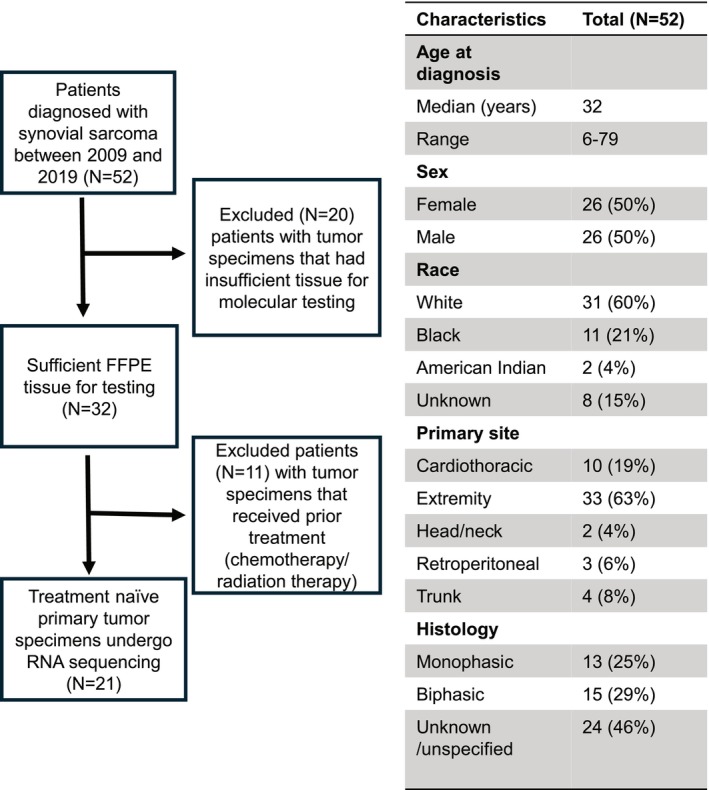
Study design, information flow and demographic summaries.

Fifty‐one unique patients with SS were diagnosed between 2009 and 2019. Half (51%) were male and the mean age was 35. Sixty percent of tumors were located on the extremities, followed by 18% trunk, 12% lung/mediastinum, 6% abdominal, and 4% head and neck. Five patients had metastasis at presentation, and an additional 11 patients developed metastases during their treatment course. Of the 52 patients, 32 had specimens available that were of sufficient quality for RNA sequencing. Twenty‐one specimens were from patients who were treatment‐naïve and therefore included in the analysis (Figure 1). All patients that were included in the nonmetastatic cohort had at least 5 years of follow‐up data that included standard surveillance imaging protocols. All patients had staging scans that were congruent with National Comprehensive Center Network (NCCN) guidelines. Specifically, 9 patients underwent whole body positron emission tomography‐computed tomography (PET/CT) and magnetic resonance imaging (MRI) of the primary site at diagnosis, and the other 12 had an MRI of the primary site and at least a CT of the chest, with the addition of CT abdomen/pelvis if clinically relevant.

Perioperative chemotherapy use was comparable between cohorts, with 50% of patients in the metastatic group receiving chemotherapy (37.5% not receiving treatment and 1 patient with unknown status) and 46% of patients in the nonmetastatic group receiving chemotherapy (46% not receiving treatment and 1 unknown). Administration of neoadjuvant or adjuvant radiation differed between cohorts. In the metastatic group, 37.5% received radiation, 25% did not, and 37.5% had unknown radiation status. In the nonmetastatic cohort, 61.5% received radiation, 30.7% did not, and 1 patient had unknown status.

Nf‐core/rnafusion, an analysis pipeline for RNA sequencing identified distinct fusion patterns between the metastatic and nonmetastatic cohorts. Among metastatic tumors, SS18–SSX1 fusions predominated (8/13; 61%), followed by SS18–SSX2 (4/13; 33%) and a single alternative NAIP–OCLN translocation (1/13; 6%). In the nonmetastatic cohort, SS18–SSX2 fusions were more frequent (4/8; 50%) compared with SS18–SSX1 (3/8; 37.5%), with one tumor harboring an alternative CTBS–GNG5 fusion (1/8; 12.5%).

**FIGURE 2:**
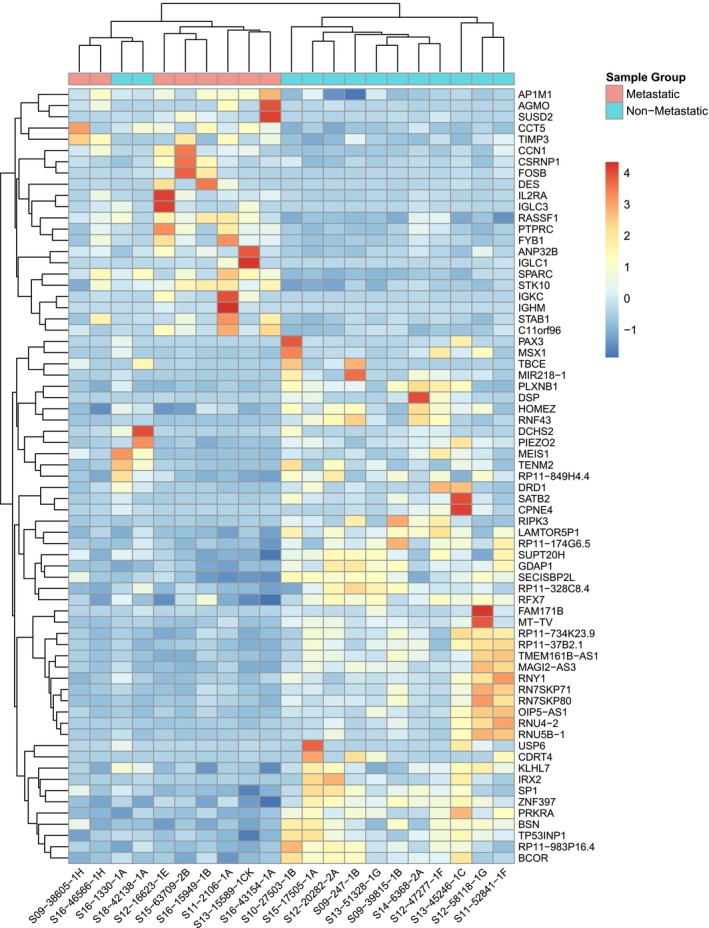
Unsupervised clustering heatmap of differentially expressed genes in untreated primary tumors, separated by cohort.

RNA sequencing showed distinctly different gene expression patterns and biological functions enriched in each cohort. Unsupervised hierarchical clustering of differentially expressed genes (FDR < 0.1) grouped samples based on expression profiles (Figure 2). Multiple pathways were enriched and overexpressed in the metastatic cohort in comparison to the nonmetastatic cohort. Primary tumors that were eventually able to metastasize demonstrated a high degree of genes expressed with oxidative phosphorylation (NES 1.60; *P* = 0.067) and epithelial to mesenchymal transition (NES 1.71; *P* = 0.025). Within the oxidative phosphorylation gene set, translocase of outer mitochondria membrane 22 (*TOMM22*), NADH: ubiquinone oxidoreductase subunit A3 (*NDUFA3*), and peptidase mitochondrial–processing subunit alpha (*PMPCA*) were all highly enriched (running ES < 0.05).

Within the subgroup of genes involved with allograft rejection, CD247 and *Spi‐1* Proto‐Oncogene (SPI1) were highly enriched (running ES < 0.05) in patients with metastatic disease. The increase in genes involved with allograft rejection may speak to the way synovial sarcoma metastases evade the immune response. CD247 is a T‐cell receptor protein that plays a role in antigen recognition in several intracellular signal‐transduction pathways. While the samples from patients who developed metastatic disease had significant or near‐significant gene expression changes, the samples from patients who did not have metastatic disease did not.

**FIGURE 3:**
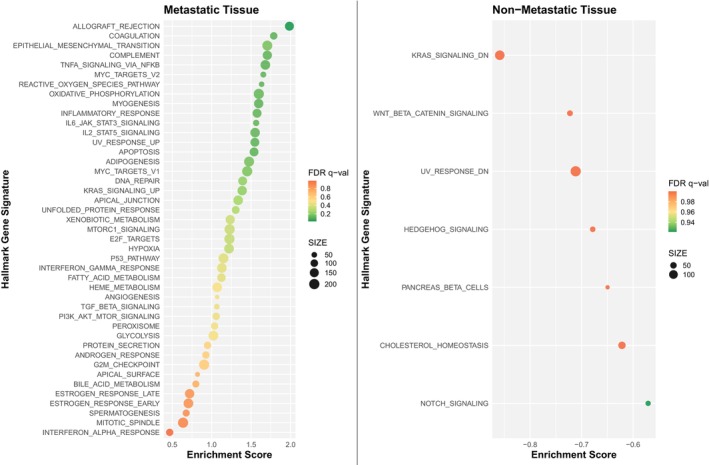
GSEA comparing metastatic and nonmetastatic samples. Of the 50 gene sets analyzed, 43 were upregulated in the metastatic group, with 18 showing statistical significance at an FDR < 25%. In contrast, only seven gene sets were upregulated in the nonmetastatic group, none of which were significant at FDR < 25%.

**FIGURE 4:**
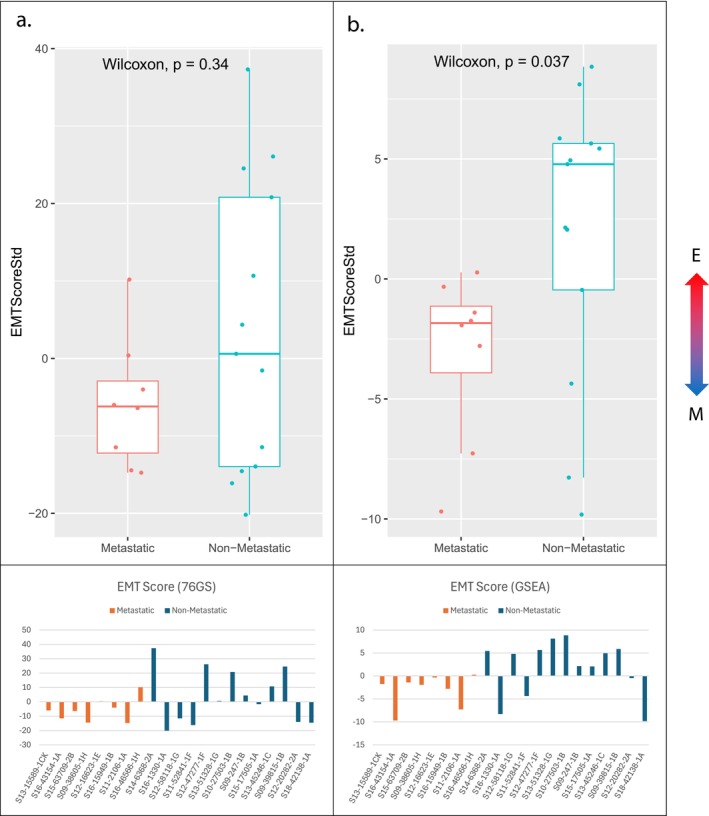
EMT scores for each cohort and sample based on (a) 76GS default method and (b) GSEA method demonstrating a more M phenotype in the metastatic group.

**FIGURE 5:**
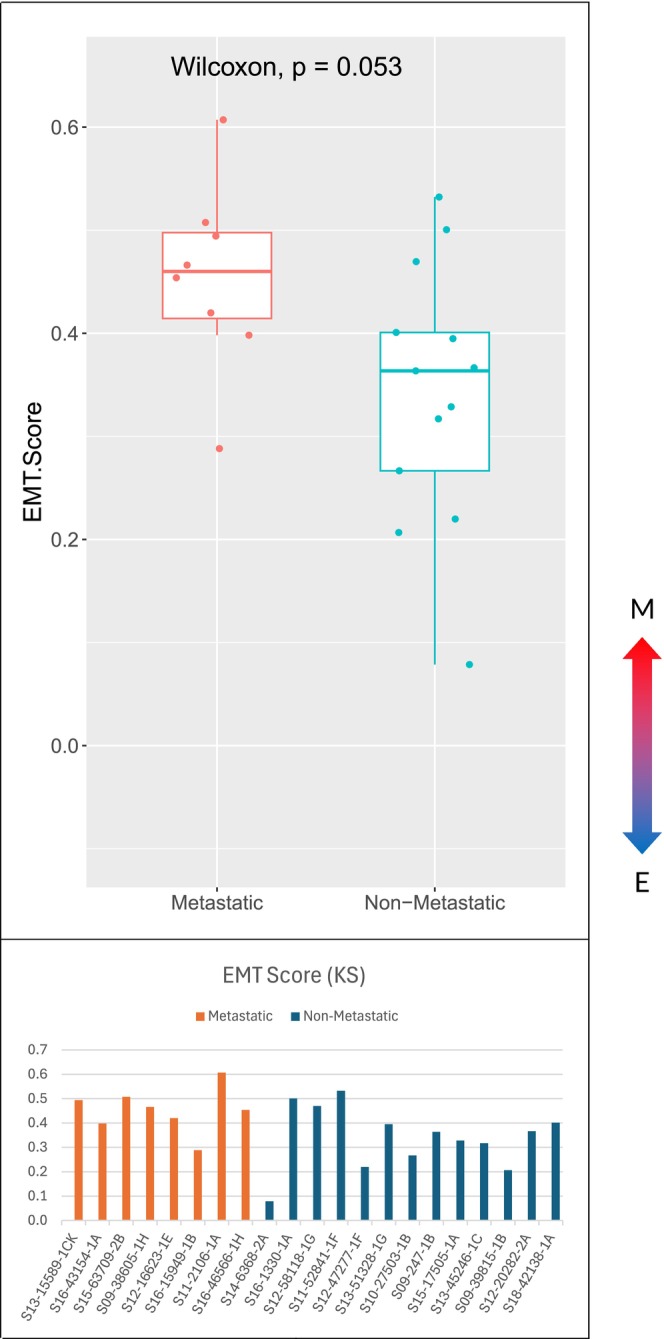
EMT scores for each cohort and sample using KS method demonstrating a more M phenotype in the metastatic group.

The Hallmark EMT gene set identified by GSEA was significantly expressed in the metastatic cohort compared to nonmetastatic (normalized enrichment score 1.71; *P* value 0.025) (Figure 3). Additionally, there was a phenotypic difference between cohorts demonstrated in all three testing methods. In the 76GS method, 52 of the 76 genes reported in the default EMT signature were expressed in our samples. Most metastatic specimens had negative EMT scores indicating mesenchymal phenotype, whereas nonmetastatic specimens had a wider distribution of scores, but they were generally more positive (epithelial) (Figure 4a). Two of the testing platforms showed primary tumors that metastasized demonstrated a more mesenchymal phenotype (GSEA Hallmark EMT gene set, *P* value 0.037 and KS method, *P* value 0.053). Although the KS method did not meet statistical significance, it trended in the same direction wherein the metastatic cohort had a more mesenchymal phenotype than the nonmetastatic cohort (Figure 5).

**FIGURE 6:**
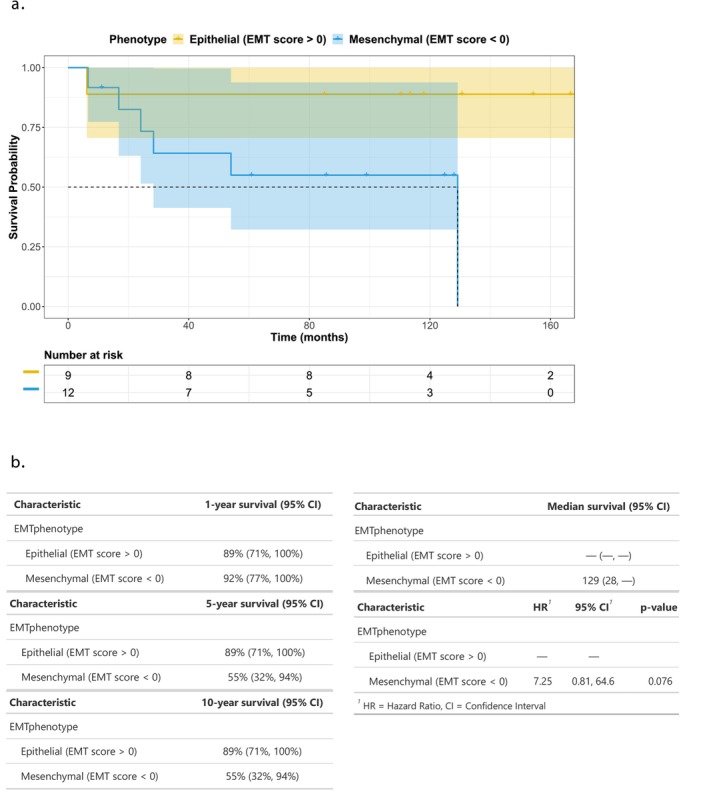
Overall survival by EMT phenotype (76GS Method) and associated survival estimates. (a) Kaplan–Meier survival curves comparing patients with epithelial phenotype (EMT score > 0, yellow) and mesenchymal phenotype (EMT score < 0, blue), with a significant difference between groups by log‐rank test (*P* = 0.044). EMT scores were calculated using the 76GS method. Shaded areas represent 95% confidence intervals. Steps in the curves represent events, whereas tick marks indicate censored observations. The number of patients at risk at selected time points is shown below the plot. (b) Summary table of survival estimates by EMT phenotype, including 1, 5, and 10 year survival probabilities with 95% confidence intervals, median survival times, and hazard ratios (HR). Median survival was 129 months for mesenchymal phenotype and not reached for epithelial phenotype. In Cox proportional hazards modeling, patients with mesenchymal phenotype exhibited an increased risk of death compared to those with epithelial phenotype (HR = 7.25; 95% CI: 0.81–64.6; *P* = 0.076).

Last, patients with a mesenchymal phenotype were consistently found to have worse outcomes with all three methods of testing (Figure 6). The log‐rank testing demonstrated χ^2^ = 4.05 (*P* = 0.04) for 76GS, and χ^2^ = 11.05 (*P* = 0.0009) for GSEA core enrichment genes. The hazard ratio for overall survival was 7.25 with a *P* value of 0.076. Although the hazard ratio trended toward worse survival, this did not reach conventional statistical significance and should be interpreted cautiously given the limited sample size.

Survival analysis was performed to evaluate the impact of EMT phenotype on overall survival. Survival time was calculated in months from the date of diagnosis to the date of last follow‐up, with patients censored if alive at last contact. Kaplan–Meier survival curves were generated for EMT‐positive and EMT‐negative groups, and differences in survival distributions were assessed using the log‐rank test, which demonstrated a significant difference between groups (*P* = 0.044; Figure 6). Additionally, a Cox proportional hazards model was fitted to estimate the hazard ratio (HR) and corresponding 95% confidence interval. EMT‐negative patients had an estimated HR of 7.25 (95% CI: 0.81–64.6; *P* = 0.076) compared to EMT‐positive patients, suggesting a potential association between mesenchymal phenotype and decreased survival.

In univariable Cox models, a mesenchymal EMT phenotype (EMT score < 0) was associated with an increased hazard of death compared with an epithelial phenotype (HR 7.25; 95% CI 0.81–64.6; *P* = 0.076). Older age at diagnosis (HR 1.06 per year; 95% CI 1.01–1.12; *P* = 0.021) and larger tumor size (HR 1.18/cm; 95% CI 1.00–1.38; *P* = 0.048) were also associated with poorer overall survival. Other demographic and clinicopathologic variables were not significantly associated with survival, and several models were unstable due to sparse events in certain categories. In the multivariable Firth‐penalized Cox model including EMT phenotype, age at diagnosis, and tumor size (complete cases, *n* = 15; 3 events), a mesenchymal EMT phenotype remained associated with a higher hazard of death (adjusted HR 7.62; 95% CI 0.17–347.1; *P* = 0.16), whereas age and tumor size were no longer statistically significant. The wide confidence intervals and loss of significance reflect the very small sample size and low number of events, and these estimates should therefore be interpreted as exploratory.

## 4 Discussion

This study showed a distinct difference in gene set enrichment between primary tumor samples from patients who did not develop metastasis during their disease course compared to those samples from patients who did develop metastatic disease. The smaller sample size of this study resulted in higher false discovery rates; however, several gene sets showed nominal enrichment signals, including oxidative phosphorylation, allograft rejection, and epithelial‐to‐mesenchymal transition (EMT). Although these gene sets met a nominal *P* value threshold of < 0.10, they should be interpreted as exploratory rather than statistically definitive, with EMT serving as the primary focus of this analysis.

To further elaborate the impacts of epithelial to mesenchymal transition, three different modalities for testing were deployed, consistently showing that the tumor samples from patients who developed metastatic disease exhibited a more mesenchymal phenotype at the time of diagnosis. Notably, patients whose tumors showed a mesenchymal phenotype based on EMT scores computed using the 76‐gene expression signature had lower overall survival. However, this observed association should be interpreted with caution due to the limited sample size and evaluated further in future studies. There were two outlier patients in the data who had more of a mesenchymal gene signature but did not develop metastatic disease. One of the patients had primary pleuropulmonary synovial sarcoma with disease along the pleural lining and invading into lung parenchyma with a disease‐specific survival of only 6 months and 19 days from diagnosis. Pleuropulmonary synovial sarcoma has a more aggressive behavior with worse outcomes than traditional synovial sarcoma [14]. The second outlier was an 11‐year‐old patient who received neoadjuvant chemotherapy for a 5 cm, grade 2 extremity tumor; despite the tumor's more mesenchymal phenotype, her other favorable prognostic features likely reduced her risk of metastasis.

SS can display a spectrum of epithelial and mesenchymal features. The fusion partner of the SS18 gene is related to the histologic subtype with the monophasic subtype more often harboring the SS18–SSX2 fusion and the biphasic subtype more often harboring SS18–SSX1 [6]. The epithelial marker E‐cadherin can be seen in the epithelial component of biphasic synovial sarcoma. E‐cadherin is a cell–cell adhesion molecule promoting structural stability and a more epithelial histology [15]. When its expression is downregulated, it leads to more mesenchymal cell structure with less cell‐to‐cell contact [16]. Epigenetic changes in SS can prevent E‐cadherin expression leading to epithelial to mesenchymal transition [17].

*CDH1* gene expression can be directly repressed by various transcriptional regulators including the proteins Snail (*SNAI1*), Zeb (*ZEB1/2*), E47 (protein product of *TCF3*), Slug (*SNAI2*), and KLF8 (*KLF8*), and indirectly repressed by proteins such as Twist (protein product of *TWIST1*), GSC (*GSC*), SIX1 (*SIX1*), E2‐2 (protein product of *TCF4*), and FOXC2 (*FOXC2*) [18]. Snail and Slug are transcription factors that bind to the promoter of *CDH1*, leading to repression of the gene [19]. In synovial sarcoma, it has been shown that the *SS18–SSX* fusion gene and its encoded proteins interact with these factors, resulting in de‐repression of *CDH1* and subsequent epithelial differentiation [17]. *SS18–SSX* does not contain a DNA‐binding domain and therefore cannot directly regulate *CDH1*. The gene product of *SS18–SSX1* interacts with Snail, whereas the gene product of *SS18–SSX2* interacts with Slug [18] These interactions have been demonstrated through co‐immunoprecipitation and chromatin immunoprecipitation assays [17]. Snail appears to be a stronger repressor of *CDH1* than Slug; thus, when Snail binding is hindered by *SS18–SSX1*, it may allow for increased epithelial differentiation, consistent with biphasic synovial sarcoma.

There are risk factors that predict worse outcomes in synovial sarcoma that are not unique to this sarcoma subtype. They include tumor grade, size, mitotic count, and degree of tumor necrosis. Molecular biomarkers have also been studied for their prognostic implications including *EZH2*, *CXCR4*, *IGF1/2*, secernin‐1 (SCRN1 protein), MMPs (matrix metalloproteinase proteins), and genes such as *CDCA2* (cell division cycle 2) and *KIF14* (kinesin family member 14). Some of these factors may be predictive of metastasis [20]. For synovial sarcoma specifically, the prognostic impact of histologic variant (monophasic vs. biphasic vs. dedifferentiated) has been explored. Thus far, results have varied. An Italian retrospective analysis of 196 patients showed that monophasic morphology portended worse outcomes [21]. Conversely, grade but not histology was found to be impactful in a large multi‐center analysis wherein 165 patients were assessed [22]. Other factors in this analysis, like larger tumor size, higher mitotic count, and tumor necrosis were also prognostic. Additionally, a multivariate analysis by European Pediatric Soft Tissue Sarcoma Study Group (EpSSG) and an analysis of one of the Cooperative Weichteilsarkom Studie (CWS) trials found no significant impact of morphology on prognosis [23, 24].

In our exploratory multivariable Cox model adjusted for age and tumor size, the mesenchymal EMT phenotype continued to show an increased hazard of death, although this association did not reach statistical significance. Given the rarity of synovial sarcoma, the small cohort size, and only three observed deaths, our multivariable estimates are imprecise and vulnerable to small‐sample bias. Nevertheless, the direction and magnitude of the effect in both univariable and multivariable analyses are consistent with our main finding that a mesenchymal EMT phenotype is associated with poorer outcomes and support the need for validation in larger, multi‐institutional cohorts.

Given the heterogeneity of soft tissue sarcoma, there has been an impetus to do subtype specific analyses such as this one. By limiting testing to one subtype of sarcoma, the number of patient samples evaluable for testing was limited. Additionally, specimen size had to be adequate for molecular profiling, and specimens exposed to prior radiation or chemotherapy were excluded, further limiting the number of patient specimens. With these limitations in mind, thought‐provoking findings were uncovered.

Synovial sarcoma has a unique pathogenesis with the translocation SS18–SSX driving oncogenic transformation through interactions with gene transcription factors. Interactions with transcription factors lead to varied degrees of transitioning between epithelial and mesenchymal characteristics. The ability to harness EMT and a more mesenchymal phenotype was associated with an increased risk of metastasis in our population as the gene set for EMT was enriched in tumors that eventually metastasized. Furthermore, synovial sarcoma samples from patients who developed metastatic disease showed a more mesenchymal gene score at the time of diagnosis in the primary tumor specimen. Our RNA expression testing was performed on the primary tumor at the time of diagnosis. If a particular gene signature, like EMT, predicts increased risk of metastasis at the time of diagnosis, it would have implications on patient care like how intensive perioperative chemotherapy and/or how rigorous surveillance should be. This finding needs to be validated in future studies but is thought‐provoking in its possible prognostic implications.

## Author Contributions

Megan Jagosky conceptualized and designed the study. Colin Anderson, Nury Steuerwald, and Jenny Chen contributed to data acquisition and analysis. Anderson O'Brien Cox, HsiTe Yang, and Guangxu Jin assisted with methodology and interpretation of results. Alicia Hamilton, Mathew Smith, Sharvil Desai, and Johann Hsu contributed to data curation and validation. Wei Zhang provided critical revisions and intellectual input. All authors participated in manuscript drafting and revision and approved the final version of the manuscript.

## Funding

This work was supported by Swim Across America and Paula Takacs Foundation.

## Ethics Statement

The data collected for this research is unique and truthfully represented. It has been exclusively submitted to this journal and is not being considered for publication elsewhere. This research was conducted with institutional review board (IRB) approval, with informed consent waived due to the retrospective nature of the study. All protected health information for the subjects involved in the study remained confidential.

## Conflicts of Interest

The authors declare no conflicts of interest.

## Data Availability

The data that supporting the findings of this study are available upon request from the corresponding author, Megan Jagosky.
